# Effectiveness of family-centred sexual health education and HPV self-sampling in promoting cervical cancer screening among hard-to-reach indian women in rural and tribal areas: a community-based pilot study

**DOI:** 10.1186/s12889-023-15602-1

**Published:** 2023-04-11

**Authors:** Mandana Vahabi, Gauravi Mishra, Sharmila Pimple, Josephine Pui-Hing Wong, Momina Khan, Vijayshree Prakash, Kavita Anand, Miya Narushima, Aisha K. Lofters

**Affiliations:** 1 Daphne Cockwell School of Nursing, Ryerson University, 4 Josephine Pui-Hing Wong, 350 Victoria Street, M5B 2K3 Toronto, ON Canada; 2 Institute for Clinical Evaluative Sciences, Toronto, ON Canada; 3 Department of Preventive Oncology, Centre for Cancer Epidemiology, Tata Memorial Centre, Homi Bhabha National Institute (HBNI), R. No. 314, 3rd Floor, Service Block, E Borges Marg, Mumbai, 400012 Maharashtra India; 4 Dalla Lana School of Public Health (Cross-appointed), University of Toronto, Toronto, ON Canada; 5 Brock University, St. Catharines, Canada; 6 Department of Family & Community Medicine, University of Toronto, St. Catharines, Canada; 7 Women’s College Hospital Research Institute, Peter Gilgan Centre for Women’s Cancers, Toronto, Canada

**Keywords:** Cervical cancer screening, Human papillomavirus self-sampling, India, Low income, Sexual health literacy, Women, Family-centered care, Rural area, Knowledge, attitudes, stigma surrounding sexually transmitted infection

## Abstract

**Background:**

While cervical cancer deaths have declined steeply in high-income countries due to the widespread use of the Papanicolaou test (Pap test), the same trend has not emerged in low or middle-income countries (LMICs). Access to screening in LMICs like India is limited due to barriers such as limited healthcare infrastructures, lack of sexual health education, and stigma demarcating sexually transmitted infections (STIs). HPV self-sampling (HPV-SS), a woman–centered and at-home method for screening, can be utilized as a unique screening tool to overcome some of these barriers. Our study examined the effectiveness of HPV-SS, supported by family-centred arts-based sexual health literacy on the uptake of cervical cancer screening among hard-to-reach women in rural and remote areas in India.

**Methods:**

Our community-based mixed methods pilot study recruited 240 participants (120 women and 120 male partners or family members) through female Accredited Social Health Activists (ASHA) across 3 Indian villages of Shirgoan, Khodala, and Jamsar in Palghar district. Inclusion criteria included women ages 30–69 who were under or never screened (UNS) and their male partners/family members aged 18 or over. Knowledge and attitudes about cervical cancer and screening and their perceived stigma surrounding STI were assessed using validated scales prior to and after attending a 2-hour arts-based sexual health education (SHE). In addition, participants’ uptake of cervical cancer screening was assessed after attendance in SHE.

**Findings:**

Results revealed significant improvement in knowledge and attitudes about cervical cancer and screening, and a reduction in the STI stigma after participation in SHE sessions (overall mean difference in Knowledge: z = 6.1 ± 2.4, P < 0.001; attitudes about Pap-test and VIA: z = 2.2 ± 8.4, P < 0.001 and z = 2.9 ± 8.2, P < 0.001; STI stigma: z = 2.8 ± 12.4, P < 0.001). 118 out of 120 female participants chose to be screened and 115 opted for HPV-SS.

**Conclusions:**

The implementation of HPV-SS coupled with family-centered arts-based and culturally appropriate SHE is highly promising in promoting cervical cancer screening among hard-to-reach women. Evidence from our study can be used to advance public health policies and inform the scale-up of similar initiatives in other villages and states across rural India and other LMICs.

## Background

Since 1948, the right to health has been acknowledged as a basic human right under Article 25 of the Universal Declaration of Human rights [1]. People’s right to the highest attainable standard of physical and mental health, including the right to health care is stressed further in Article 12 of the International Covenant on Economic, Social and Cultural Rights [2]. The right to health implies having free access to health care, and it includes the right to health information and education as well as the right to prevention, treatment, and control of disease [1]. However, many groups around the world are deprived of this right and women living in low- and middle-income countries (LMICs) are one such example. Roughly 90% of the global burden of cervical cancer deaths occurs among women in LMICs and almost all cases are linked to human papillomavirus (HPV), the most common sexually transmitted infection (STI) [3, 4] With its pre-invasive phase generally lasting for 10–15 years, cervical cancer is highly preventable through early detection and treatment [5]. However, India accounts for roughly 20% of the worldwide cases and it is the second leading cause of cancer death for Indian women, particularly in younger women who are in the prime of their lives [6, 7]. Although cervical cancer deaths have declined steadily and steeply in high-income countries (HIC) due to the widespread use of the Pap test, a test which checks for abnormalities in the cells of the cervix, the same trend has not been observed in LMICs. Access to this preventive measure is highly intertwined with a functioning health system, equipped with trained gynecologists/health care personnel, laboratory infrastructure, and screening programs [8–10] All of these are necessary for the elimination of cervical cancer. Unfortunately, neither the Pap test nor the Visual Inspection with Acetic Acid (VIA) test (an alternative method of cervical cancer screening) is easily accessible to many women living in LMICs due to the aforementioned limited infrastructure along with out-of-pocket costs, cultural sensitivities, and lack of female healthcare providers [8–10]. The coverage of cervical cancer screening in LMICs like India is only 19% compared to 63% in HIC [11]. Hence women who do not have access to cervical cancer screening are deprived of their basic human rights to health and health care. The low uptake of screening in India has been attributed to limited knowledge, awareness and availability of cervical cancer and screening programs, and stigma surrounding sexually transmitted infections (STIs) including HPV (the primary cause of cervical cancer) [12, 13].

HPV self-sampling (HPV-SS) along with family centered sexual health education which include not only women, but also their male partners or adult male family members may serve as a unique approach to overcome some of these barriers. By using HPV-SS, women can self-collect samples in the privacy of their homes without requiring pelvic examinations. With similar sensitivity and specificity to traditional screening methods, HPV-SS is also cost-effective which increases scale-up efficacy for populations that are hard to reach [14, 15]. Studies among South Asian women in HICs have also found HPV-SS to be widely accepted compared to traditional screening approaches as it aligned with women’s cultural norms and values that highly stress modesty and privacy [16, 17]. Through gender specific sexual health education, barriers to HPV screening may be reduced further by decreasing social stigma related to STIs [18, 19]. Involving women’s male partners or male family members contributes to increased open dialogue and increased health literacy on HPV and cancer screening within the family as well as in the community, which in turn facilitate women’s participation to undertake screening. Evidence suggests that involvement of male partners in sexual and preventive health care is an effective strategy in women’s health outcomes and acceptable to women [20–24]. The World Health Organization also recommends men’s engagement in the prevention of cervical cancer in LMICs [24]. Through family-based arts-based sexual health education, barriers to HPV screening can be reduced further by decreasing social stigma related to STIs [25].

Our study “Prevention of Cervical Cancer in India through Self-Sampling” (PCCIS) aimed to increase the uptake of cervical cancer screening among under or never screened (UNS) Indian women in rural areas through HPV Self- Sampling (HPV-SS) supported by family centered, arts-based sexual health literacy intervention. We hypothesized that after participation in sexual health education sessions (SHEs) there would be (1) an increase in sexual health literacy and a reduction in stigma related to cervical cancer and screening; and (2) a higher uptake of HPV self-sampling compared to traditional method of screening (Pap or VIA test) among UNS women.

## Methods

### Design

A community- based mixed methods design was used to assess the effectiveness of our novel and innovative cervical cancer screening program. The program offered HPV-SS supported with arts-based SHE sessions for both women and men to promote community awareness of cervical cancer and screening. The study aimed to assess the impact of the program on participants’ knowledge, attitudes, stigma related to cervical cancer and screening and their uptake of cervical cancer screening using HPV-SS. Participant’s perceived challenges and facilitators in utilizing cervical cancer screening and their views about HPV-SS were also explored. This paper focuses only on quantitative findings related to the effectiveness of the proposed program.

### Setting

**Fig. 1:**
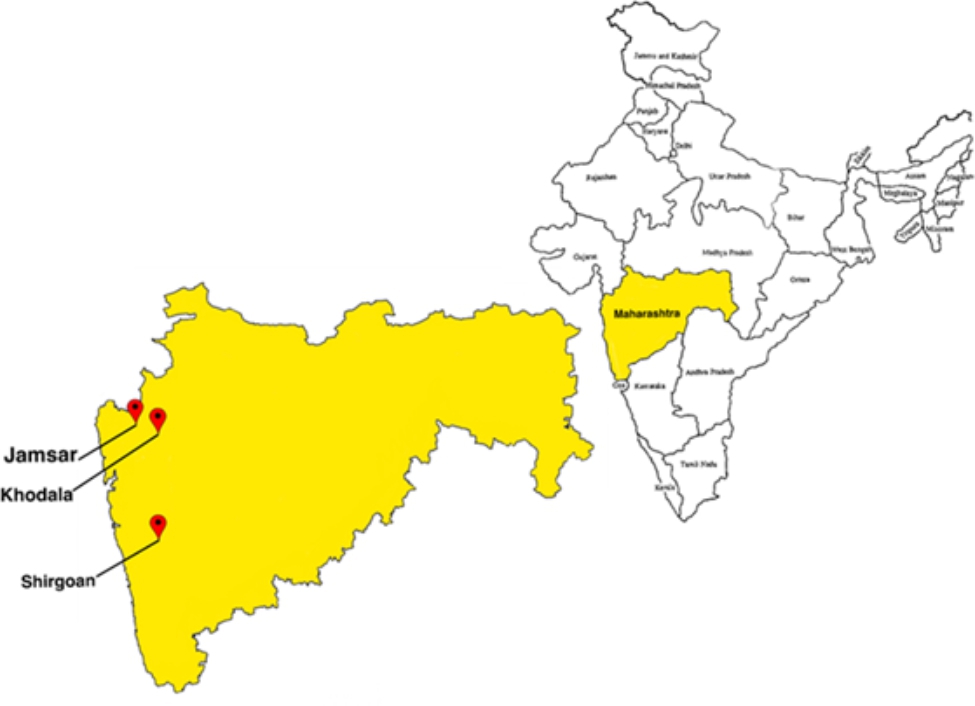
Study setting (i.e., Geographic location the 3 selected rural and tribal villages in India)

The project was implemented in three rural villages (Shirgoan in Palghar taluka (taluka = group of villages), Khodala in Mokhada and Jamsar in Jawhar) in the Palghar district of Maharashtra India (Fig. 1).

Jawhar and Mokhada talukas, which contribute to 91.6% and 92.1% of the tribal population respectively, have hard-to-reach hilly tribal regions which makes access a challenge. Shirgaon, despite being a rural area has better roadway access compared to the other two villages. All these 3 villages were considered the most underserved in terms of cancer screening and general health care. Considering the lack of literature about cervical cancer screening rates in these areas, an environmental scan through door-to-door surveys was conducted by our research team (see Table 1). The results confirmed low cervical cancer screening rates of 2% in Shirgoan, < 1% in Khodala, and 0% in Jamsar, low knowledge about cervical cancer, and a high rate of cancer-related risk factors. It is important to note that although the literacy rates were high for adult residents in these villages, the quality of formal education is reported poor due to a lack of resources and challenges in commuting to schools, especially for Jamsar and Khodala. The schooling facilities are not up to standard across these villages. All the children, irrespective of their age and class, are made to sit in the same classroom and are taught by the same teacher. Due to a lack of standard education, boys and girls completing secondary education cannot even write their names or read properly. The prevalence of cancer-related risk factors like the use of tobacco or alcohol is high for males, however, under-reporting is common due to the stigma associated with these behaviours. This may underestimate the true prevalence of risk factors for men and women in these villages. Furthermore, no cervical cancer screening facilities exist at any nearby private clinics or Government Primary Health Centers (PHCs) in these areas.

**Table 1:** Environmental scan- Primary data

	Taluka	Shirgoan	Mokhada	Jawhar
	Villages (implementation)	Shirgaon N (%)	Khodala N (%)	Jamsar N (%)
1.	Adult male population	939 (48.75)	582 (47.43)	593 (48.80)
2.	Adult female population	987 (51.24)	645 (52.56)	622 (51.19)
3.	Adult male literacy	879 (93.61)	511(87.80)	399 (67.28)
4.	Adult female literacy	833 (84.40)	454(70.39)	322(51.77)
5.	Prevalence of Alcohol in male	164 (17.47)	131 (22.51)	199 (33.56)
6	Prevalence of Alcohol in female	10 (1.01)	15 (2.33)	43 (6.91)
7	Prevalence of Tobacco in males	175 (18.64)	216 (37.11)	237 (39.97)
8	Prevalence of Tobacco in female	57 (5.78)	193 (29.92)	113 (18.17)
9	Heard about cervical cancer - males	60 (6.39)	7 (1.20)	2 (0.34)
10	Heard about cervical cancer - female	140 (14.18)	15 (2.33)	5 (0.80)
11	Population in age 30–69 female	694 (70.31)	401 (62.17)	394 (63.34)
12	Cervical screening uptakes in past*	**19 (1.93)**	**2 (0.31)**	**0 (0)**
13	Availability of cervical cancer screening tests at Primary Health care Centre.	Nil	Nil	Nil
15	Distance to Rural Hospital	10 KM	24 KM	24 KM

### Sample size. Inclusion criteria, and recruitment strategies

A Purposive sample of 240 women and men (120 women and 120 male partners/family members) were recruited across the 3 villages (80 per village (i.e., 40 women and 40 men)) through our female community champions along with our male and female medical social health care personnel (medically trained staff). The community champions were trusted members of our selected communities and were part of a new model of care that was adopted by the Indian government called local accredited social health activists (ASHA, meaning hope). The ASHA program relies on building capacity in local and remote areas by recruiting trusted community members and training them to promote awareness about the health-related issues facing their respective communities.

The inclusion criteria for female participants included (a) aged 30–69 years, (b) under or never screened (i.e., self-report of > 4 years since last Pap test/VIA including no history of Pap test/VIA); (c) had ever been sexually active; (d) was able to provide informed consent; and (e) was willing to share contact information with the study team. Male participants were selected by female participants who considered them as “supportive” men in their life or social networks (e.g., spouse, father, brother, cousin, son). The male participants were required to be 18 years and older.

Our study protocol received approval from ethics review boards (REB) of all participating institutes: Toronto Metropolitan University (formerly known as Ryerson University; reference number: REB 2020 − 104), Tata Memorial Center (reference number: OIEC/3786/2021 /00003), the Women’s College Hospital (REB # 2021-0019-B), Brock University (REB # 20–362 – VAHABI), and University of Alberta (REB # Pro00109894).

### Study intervention and implementation

All study staff received three full-days training about the project activities, cervical cancer and screening and study protocols (i.e., community outreach, participants’ eligibility criteria, obtaining consent form, study questionnaires and focus groups). All data were collected in face-to-face interviews by either our research assistants (RAs) or medically trained staff either in Hindi or Marathi (the spoken languages across our 3 selected villages). Our female community champions (i.e., ASHA workers) along with our medical social health care personnel identified and approached women in their respective villages. Our female medical social staff assessed eligibility, explained and obtained consent, and conducted interview-based pre survey questionnaire with women participants. Each woman participant identified a supportive male partner or male family member. If the supportive male partner was present our male medical social health care worker assessed their eligibility, obtained their consent forms, and conducted the interview based pre survey questionnaire. If the supportive male partner was not present, contact information was collected and the person was contacted at a later time by our male staff and a similar process as mentioned above was followed.

The woman and her male support were then invited to the study camp site in their respective villages where they attended the gender-specific arts-based SHE sessions separately, completed the interviewer-administered pre- and post-SHE session survey questionnaires, attended a movie matinee (described below), and decided to whether or not undergo cervical cancer screening and if so what kind of screening method to utilize (i.e., Pap/VIA/HPV-SS). Each SHE session was approximately 90 min in length and in order to prevent COVID- 19 transmission, consisted of a small group of 3–5 participants with two project medically trained team members, and 1 research assistant. Topics included HPV virus in the contexts of STIs, the stigma surrounding cervical cancer, method of transmission, male and female cancers resulting from HPV virus, risk factors of cervical cancer, signs and symptoms of cervical cancer and importance of screening for early identification of the disease. Considering that some male partners or male family members were working during the day we also offered individual SHE sessions as needed.

After completing the post-SHE session survey questionnaire, which took about 30 min to complete, all male and female participants regrouped and attended a 30-minute Movie Matinee in Hindi that was offered on the same day as the SHE. Contents of the video for the Movie Matinee included stories of two women – one who died of cervical cancer and the impact of her death on her family’s wellbeing; and one who was screened, diagnosed, and received treatment. The movie dispelled misconceptions and stigmas surrounding sexually transmitted infections (STIs), HPV and cervical cancer, and how to address them. The video was followed by a 5-minutes HPV-SS demonstration video in Hindi, and 2-3- minute testimonial clips: two testimonials from physicians about the importance of cervical cancer screening and the benefits of HPV-SS + + and three testimonials of cervical cancer survivors and their children promoting the importance of cervical cancer screening.

All participants were then surveyed on their intention to take part in cervical cancer screening. They were given 3 coloured paper-ribbons with pre-printed potential participation codes when they arrived at the camp site (Red = no, Green = yes to HPV-SS, and Orange = prefer to undergo PAP or VIA) to represent the women’s and their male partners’ decision to participate in screening. Participants dropped their ribbons into the colour coded boxes representing those 3 choices at the end of the show and received their preferred cervical cancer screening methods. Collected samples were mailed to the designated hospital labs for processing. Those who tested negative for HPV/Pap/VIA were informed by their respective study female health professional, and their name was included in a database that will be maintained at TATA memorial center (TMC) to be contacted again for screening in 3 years (if they chose Pap/Via) or 5 years (if they chose HPV-SS.) Those who tested positive received a VIA or Pap test by our study research primary health care providers who were trained by our TMC collaborators. Medical follow-up tests and treatments were arranged through TMC for those with positive pap-tests.

Packed Food/refreshments was served at SHE sessions and participants were compensated for their time. Moreover, the SHE and the Movie Matinee were held in accordance with the local Covid guidelines by holding small groups, ensuring proper social distancing, wearing masks and PPE by all attendees and staff.

### Data Collection

All consenting women and men participants completed an interviewer-administered questionnaire before and after the SHE sessions. Both survey questionnaires were translated in Hindi and Marathi and then back-translated into English by two independent bilingual researchers. The accuracy of the translated version was pilot tested for flow, clarity and comprehension through personal interviews with five Indian women.

The pre survey questionnaire, which took 45 min to complete, included items adopted from validated scales on socioeconomic characteristics like age, marital status, occupation, and income, medical history, knowledge and attitudes about HPV, cervical cancer and screening, and perceived cultural stigma about cervical cancer [26–31]. The post questionnaire which took 30 min to complete excluded items on sociodemographic and medical history but kept the remaining components. There were 10 questions on knowledge of cervical cancer, 6 questions on knowledge of HPV and 9 questions on knowledge of cervical cancer screening. The questionnaire also included 23 questions on attitudes toward cervical screening and 10 questions on stigma surrounding STIs.

Participants’ decision to undertake cervical cancer screening and the type of cervical cancer screening chosen was collected. Results of participants’ primary screening test, follow-up, treatment were compiled.

### Analysis

All statistical analysis was performed using IBM SPSS version 28. Univariate descriptive statistics was used to profile the study participants based on survey responses. For knowledge and attitudes about cervical cancer and screening the overall score was calculated by taking the sum of correct responses wherein the “correct responses” were assigned as 1 and “incorrect responses” and “do not know” response were assigned as 0. Thus, the total score of each participant ranged between 0 and 10 for cervical cancer knowledge, 0–6 for HPV knowledge, and 0–9 for cervical cancer screening knowledge.

For cervical cancer screening attitudes (PAP & VIA Test), and stigma surrounding sexually transmitted infections (STIs), either a 4 or 5-point Likert scale were used across all domains. The scores were assigned according to participants’ assessment of the statement. The higher the score the more negative the attitude/ stigma towards the question/statement posed. The overall score was calculated by taking the sum of the question’s responses where the “strongly disagree” was assigned as 1 while the “strongly agree” was assigned as 5. However, there were certain questions/statements which were framed in a manner where the order was reversed to capture the attitude accordingly. Thus, the total score of each participant ranged between 23 and 115 for cervical screening attitudes and between 10 and 50 for stigma surrounding STIs.

The overall score for each domain was presented as mean, standard deviation, minimum and maximum values. The change in scores from pre-intervention to post-intervention was compared using Wilcoxon signed rank test for overall participants as well as for female and male participants. The comparison of mean difference in scores at pre-intervention, post-intervention and its change from pre- to post-intervention between males and females was done using Mann Whitney U test. All the tests were two-sided and p value < 0.05 was considered statistically significant.

## Results

### Participants’ sociodemographic and clinical characteristics

As presented in Table 2, of the 240 participants (40 women, 40 men from each of the 3villages) the average age of participants was 43. The majority (70%) had no education or only completed elementary school. Half of the participants reported their family income as not enough or just barely meeting their family needs, and 90% did not have medical insurance. Most participants were married with 3 or more children and almost all (96%) had never received any kind of sexual health education prior to our study. 79% of participants considered it improbable or unlikely for a woman to get cervical cancer. About 78% of women participants either considered themselves at no risk or very small risk of getting cervical cancer. Interestingly all male participants (100%) believed their female partners to be at no risk or small risk of getting cervical cancer. Nearly 7% of participants reported having a family history of cancer. Only 3 had a family history of cervical cancer and the rest reported having a family history of breast, colon and other types of cancer such as liver, esophagus, and prostate.

**Table 2:** Socio-demographic Characteristics of Study Participants

Characteristic	Overall N = 240	Female N = 120	Male N = 120
**Village**			
Shirgoan	80 (33.3%)	40 (33.3%)	40 (33.3%)
Khodala	80 (33.3%)	40 (33.3%)	40 (33.3%)
Jamsar	80 (33.3%)	40 (33.3%)	40 (33.3%)
**Age**			
20–30	8 (3.3%)	5 (4.2%)	3 (2.5%)
31–40	102 (42.5%)	64 (53.3%)	38 (31.7%)
41–50	78 (32.5%)	34 (28.3%)	44 (36.7%)
51–60	44 (18.3%)	17 (14.2%)	27 (22.5%)
61–70	8 (3.3%)	0 (0%)	8 (6.7%)
Mean	43.3	41	45.6
Median	42	39	45
Mode	35	35	45
Range (min-max)	23–70	30–60	23–70
**Education**			
No Schooling	47(19.6%)	31 (25.8%)	16 (13.3%)
Some Primary School	47 (19.6%)	28 (23.3%)	19 (15.8%)
Primary School Complete	28 (11.7%)	13 (10.8%)	15 (12.5%)
Some Secondary School	49 (20.4%)	22 (18.3%)	27 (22.5%)
Secondary School Complete	34 (14.2%)	10 (8.3%)	24 (20%)
Junior College/Some University	12 (5.0%)	2 (1.7%)	10 (8.3%)
Senior College(University Complete)	10 (4.2%)	2 (1.7%)	8 (6.7%)
Graduate Degree	13 (5.4%)	12 (10%)	1 (0.8%)
**Occupation**			
Govt Employee	6 (2.5%)	1 (0.8%)	5 (4.2%)
Non-Govt Employee	34 (14.2%)	7 (5.8%)	27 (22.5%)
Self Employed	125 (52.1%)	46 (38.3%)	79 (65.8%)
Home maker	66 (27.5%)	66 (55%)	0 (0%)
Retired	6 (2.5%)	0 (0%)	6 (5%)
Unemployed Able To Work	1 (0.4%)	0 (0%)	1 (0.8%)
Others	2(0.8%)	0 (0%)	2 (1.7%)
**Income**			
Well Off	38 (15.8%)	9 (7.5%)	29 (24.2%)
Living Comfortably	84 (35%)	53 (44.2%)	31 (25.8%)
Just Getting By	94 (39.2%)	35 (29.2%)	59 (49.2%)
Not Enough to Meet Family Needs	24 (10%)	23 (19.2%)	1 (0.8%)
**Marital Status**			
Single/Unmarried	1 (0.4%)	0 (0%)	1 (0.8%)
Married	238 (99.2%)	119 (99.2%)	119 (99.2%)
Widowed	1 (0.4%)	1 (0.8%)	0 (0%)
**Number of children**			
1	40 (17.2%)	19 (15.8%)	21(18.1%)
2	91 (39.1%)	46 (38.3%)	45 (38.8%)
3	52 (22.3%)	27 (22.5%)	25 (21.6%)
4	35 (15%)	17 (14.2%)	18(15.5%)
5	11 (4.7%)	6 (5%)	5 (4.3%)
6	4 (1.7%)	2 (1.7%)	2 (1.7%)
**Perceived risk of a woman to get cervical cancer**			
Not at all likely	189 (78.8%)	81 (67.5%)	108 (90%)
Slightly likely	25 (10%)	13 (10.8%)	12 (10%)
Moderately likely	6 (2.5%)	6 (5%)	0 (0%)
Very likely	20 (8.3%)	20 (16.7%)	0 (0%)
**Received any sexual health education prior to our study**			
Yes	11 (4.6%)	6 (5%)	5 (4.2%)
No	229 (95.4%)	114 (95%)	115 (95.8%)

### Total # of sexual health educational sessions (SHE) delivered

One hundred fifty-three sexual health education (SHE) sessions were delivered. Sixty-one SHE sessions were delivered by our female medical social health care personnel to 120 women participants across the 3 selected rural villages and 92 SHE sessions were delivered by our male medical social health care personnel to 120 supportive male partners of women participants either in small group or one-on-one session.

### Change in Participants’ knowledge, attitudes and stigma surrounding cervical cancer and screening after participation in SHE sessions

We found a significant increase in participants’ knowledge and attitudes about cervical cancer and screening after participation in SHE sessions (Tables 3 and 4). Table 3 demonstrates that all 120 women along with their male partners (i.e., 120 males) demonstrated a significant increase in knowledge of cervical cancer, HPV infection as a cause for cervical cancer and transmission of infection, PAP and VIA tests (i.e. z = 6.13 ± 2.44, P < 0.001; z = 4.32 ± 1.20, P < 0.001; z = 6.03 ± 1.91, P < 0.001; z = 5.45 ± 1.9, P < 0.001 respectively). The overall mean score change in knowledge of Pap-test (pre-intervention to post intervention) between females and male participants increased significantly. Women’s knowledge about Pap-test after their participation in SHE sessions improved more than men’s (z = 6.30 ± 2.02 vs. z = 5.75 ± 1.76, p < 0.001).

**Table 3:** Improved knowledge on HPV, cervical cancer overall, and Pap-test and VIA screening

Indicator	Gender	Pre Intervention		Post Intervention		Mean change ± SD	p value (pre-post)	
		Mean ± SD	Min – Max	Mean ± SD	Min – Max			
**Knowledge about HPV infection, by gender**	Overall	0.08 ± 0.356	0–4	4.40 ± 1.175	0–6	4.321 ± 1.207	< 0.001**	A significant difference in mean HPV knowledge score was observed across male and female participants
	Female	0.13 ± 0.466	0–4	4.49 ± 1.414	0–6	4.358 ± 1.46	< 0.001**	
	Male	0.03 ± 0.18	0–1	4.32 ± 0.869	0–6	4.283 ± 0.891	< 0.001**	
**Knowledge of cervical cancer overall and by gender**	Overall	0.19 ± 0.618	0–4	6.32 ± 2.434	0–10	6.129 ± 2.442	< 0.001**	No significant difference in mean knowledge score was observed across male and female participants.
	Female	0.26 ± 0.728	0–4	6.46 ± 2.704	1–10	6.200 ± 2.727	< 0.001**	
	Male	0.13 ± 0.477	0–3	6.18 ± 2.134	0–10	6.058 ± 2.127	< 0.001**	
**Knowledge about Pap-test as a method of cervical cancer screening by gender**	Overall	0.29 ± 0.75	0–6	6.32 ± 1.76	0–9	6.03 ± 1.91	< 0.001**	The overall change in mean score Pap-test (pre-intervention to post intervention) between females and male participants increased significantly. Women’s knowledge about Pap-test after their participation in SHE sessions improved more than men’s.
	Female	0.13 ± 0.46	0–3	6.43 ± 1.93	1–9	6.30 ± 2.02	< 0.001**	
	Male	0.46 ± 0.93	0–6	6.21 ± 1.56	0–9	5.75 ± 1.76	< 0.001**	
**Knowledge about Visual inspection with acetic acid (VIA) as a method of cervical cancer screening by gender**	Overall	0.30 ± 0.79	0–6	5.75 ± 1.64	0–9	5.45 ± 1.91	< 0.001**	The overall change in mean score Pap-test (pre-intervention to post-intervention) between female and male participants increased significantly
	Female	0.08 ± 0.35	0–2	6.02 ± 1.70	2–9	5.94 ± 1.76	< 0.001**	
	Male	0.53 ± 1.01	0–6	5.49 ± 1.55	0–8	4.96 ± 1.93	< 0.001**	

Test Used: Wilcoxon Signed Rank Test; ** signifies highly significant p value < 0.05

We found an improvement in attitudes towards Pap tests among the participants overall (Table 4), as shown by a decline in mean score from pre-intervention to post-intervention. This finding seemed to be driven by the men participants (z=-2.20 ± 8.35, p < 0.001; z=-6.13 ± 6.40, p < 0.001 respectively), as the opposite was observed among female participants (i.e., an elevation in female mean scores which meant a deterioration in their attitudes i.e., z = 1.73 ± 8.23, p = 0.002). No significant difference in attitudes towards Pap test was observed between female and male participants prior to attendance in SHE sessions. However, the improvement in attitudes was significantly higher among men than women after participation in SHE. Similarly, a decline in mean scores attitude toward VIA was noted from pre-intervention to post-intervention which was witnessed across all the participants and among men participants (z=-2.93 ± 8.17, p < 00.1; z=-7.03 ± 5.92, p < 0.001 respectively). However, the opposite was observed among female participants (i.e., a significant elevation in mean scores, i.e., z = 1.18 ± 8.06, p = 0.013). The improvement in attitudes was significantly higher among men than women.

**Table 4:** Improved Attitudes towards Pap-test and VIA screening

Indicator	Gender	Pre Intervention		Post Intervention		Mean change ± SD	p value (pre-post)	
		Mean ± SD	Min – Max	Mean ± SD	Min – Max			
**Attitudes towards Pap smear as a screening test after participating in the study.**	Overall	70.60 ± 4.56	58–86	68.39 ± 7.24	51–93	-2.20 ± 8.35	< 0.001**	No significant difference was observed prior to attendance in SHE session between female and male participants. However, after participation in SHE there was significant improvement among men compared to women participants
	Female	70.63 ± 4.80	63–89	72.36 ± 5.42	51–93	1.73 ± 8.23	0.002*	
	Male	70.56 ± 4.33	63–89	64.43 ± 5.42	58–86	-6.13 ± 6.40	< 0.001**	
**Attitudes towards Visual inspection with acetic acid (VIA) as a screening test after participating in the study.**	Overall	70.69 ± 4.897	64–104	67.77 ± 7.099	46–89	-2.925 ± 8.172	< 0.001**	A significant difference in attitudes towards VIA was observed across male and female participants. Women’s attitudes toward the VIA test improved more than men’s.
	Female	70.71 ± 4.484	66–86	71.89 ± 6.694	50–89	1.183 ± 8.064	0.013	
	Male	70.68 ± 5.297	64–104	63.64 ± 4.701	46–73	-7.033 ± 5.921	< 0.001**	

Test Used: Wilcoxon Signed Rank Test; ** signifies highly significant p value < 0.05; * signifies significant p value < 0.05

Table 5 also demonstrates a significant decrease in stigma surrounding STIs including HPV. A lower mean score means a better attitude toward stigmatization of cervical cancer. For overall participants there was a significant reduction in stigma post intervention (i.e., mean scores of 34 pre intervention vs. 31 post intervention, mean score change z=-2.77 ± 12.45, p < 0.001). The reduction in stigma surrounding STIs was significantly more apparent in male partners than women participants after participation in SHE (post intervention mean scores for female z = 36.14 ± 12.06 vs. male 26.43 ± 8.06, p < 0.001).

**Table 5:** Change in Stigma Surrounding STIs

	Pre Intervention		Post Intervention		Mean change ± SD	p value (pre-post)
	Mean ± SD	Min - Max	Mean ± SD	Min - Max		
**Overall**	34.05 ± 10.79	10–50	31.28 ± 11.332	10–50	-2.767 ± 12.446	< 0.001**
**Female**	37.31 ± 12.19	10–50	36.14 ± 12.06	10–50	-1.167 ± 15.163	0.345
**Male**	30.79 ± 7.987	12–45	26.43 ± 8.055	12–46	-4.367 ± 8.718	< 0.001**

Test Used: Wilcoxon Signed Rank Test; ** signifies highly significant p value < 0.05

### Type of cervical cancer screening method by age-group

Table 6 demonstrates that among 120 women enrolled in the study 115 accepted HPV-SS as the primary screening test, 2 opted for Pap and 1 opted for VIA testing. 2 women refused to be screened.

**Table 6:** Type of cervical cancer screening method by age

Age	30–40 yrs	41–50 yrs	Above 51	Total
HPV-SS	64	34	17	115
Pap	2	0	0	2
VIA	1	0	0	1

### Positive test results detected by screening method across three villages

Table 7 explicates that among 115 women enrolled in the study who accepted HPV-SS, 10 tested positive. There was no positive test result among those who opted for PAP or VIA. Among the 10 screen-positive women on the HPV test, 9 women underwent Pap as a triage test, while 1 woman refused the Pap-test. Among the 9 women who underwent Pap as a triage test, 4 women were detected with an abnormal report and were referred to colposcopy. Follow-up among 4 women who had undergone colposcopy found that only one woman had an abnormal lesion. Her histopathology showed a high-grade lesion (CIN-3) and she underwent Loop electrosurgical excision of the lesion (treatment). Women who were negative on colposcopy were advised to follow-up screening after 1 year. Women negative on HPV-SS as a primary screening test were advised to have a follow-up screening after 5 years, and women negative on Pap/VIA test were advised to follow-up after 3 years.

**Table 7:** Screening test results by type of screening for women across 3 selected villages

	Villages			
	Shirgoan	Khodala	Jamsar	Total
**1. Women opted for HPV-SS ( N = 115)**				
a. HPV test negative	36	32	37	105
b. HPV test result positive	1	6	3	10
**2. Pap test triage test advised (N = 10)**				
a. Loss to follow up	0	0	1	1
b. Pap test done	1	6	2	9
**3. Pap test results (N = 9)**				
a. Normal	0	4	1	5
b. Abnormal	1	2	1	4
**4. Results of Colposcopy test done for women with abnormal Pap (N = 4)**				
a. Normal	1	2	0	3
b. Abnormal	0	0	1	1
**5. Precancerous Lesion detected**	0	0	1	1

## Discussion

To our knowledge, this is the first study to explore the effectiveness of HPV-SS supported by arts-based sexual health education in promoting cervical cancer screening uptake among low-income rural women in India. Our study found limited knowledge and negative attitudes about cervical cancer and screening not only among rural women but also their male partners. Furthermore, we found that most of our participants had never received any sexual health education in their lifetime. Interestingly, the majority of our study participants considered the risk of getting cervical cancer as improbable or quite unlikely.

Our findings are consistent with prior research in lower-middle income countries [32, 33]. Existing evidence demonstrate that women in rural India have a high risk of developing cervical cancer due to young age at marriage, low literacy, low screening uptake associated with structural barriers such as lack of access to sexual health information, long distance from healthcare facilities, insufficient available healthcare providers, and persistence of genital infections, including HPV and other STI [34, 35]. However, despite these risk factors, Indian women and their male partners in rural areas were completely unaware of the threat of cervical cancer and the importance of its early identification and treatment. These are a clear indication of violation of their basic human rights to health as indicated under Article 25 of the Universal Declaration of Human rights [1]. The right to health includes access to health information and education as well as the right to prevention, treatment, and control of disease [1].

Lack of education and misinformation surrounding HPV and cervical cancer among women and men is a significant barrier to accessing screening for early detection and prevention. Our study showed a significant increase in knowledge and improvement in attitudes about cervical cancer and screening following participation in our arts-based sexual health education sessions among all 240 participants. These findings are consistent with interactive arts-based strategies such as audio and theater programs offered to deliver sexual health education focusing on HIV in Tamil Nadu, India. Nambiar and colleagues found that those who had exposure to the sexual health education programs had significantly higher knowledge on HIV and its therapies as well as a significantly higher propensity to ask doctors questions about it [36]. Similarly, in the rural area of Namkum and Kanke in India, Akhorui and colleagues found that reproductive and sexual health information delivered through audio-visual materials and group discussions showed significantly greater knowledge and improvement in health practises among tribal women [37]. Our story-telling sexual health education approach has been proven to be effective in promoting sexual health literacy [38]. Storytelling is an integral aspect of everyday life. Stories enable participants to engage with the contents in ways that they are able to make sense in the context of their own lives, through self-reflection and dialogue with others [39, 40]. They are also effective in reducing stigma and engaging the audience emotively to promote empathy, openness and positive action for health [41–44]. Storytelling is a well-established and well-accepted means of cultural learning for empowerment in India [45], particularly among women who have been historically left out of more formal learning institutions [46].

Our findings supported our central hypotheses of higher knowledge, improvement in attitudes, and reduction in the stigma surrounding cervical cancer and screening after participation in arts based sexual health education sessions (SHEs). Although a significant improvement in attitudes and reduction in stigma surrounding cervical cancer and screening prevailed among male participants, a similar trend was not observed for female participants. The decrease in attitudes toward cervical cancer may be related to the social expectation for Indian women to be modest; worries about cervical cancer, and discomfort in viewing women’s genitals in the SHE materials on HPV screening. Furthermore, women may be more vulnerable to STI stigma since women with STIs have been accused of and condemned for having “loose character” and viewed as “vectors of disease” who pass their infection on to men. Hence, they are at higher risk of being stigmatized due to entrenched gender norms and stereotypes associated with these infections. Internalized, anticipated, perceived, enacted, and secondary stigmas surrounding HPV, a STI and primary cause of cervical cancer, may deter uptake of cervical cancer screening due to disgust/shame, fear of diagnosis, entrenched gender norms, and negative stereotypes associated with infections [47, 48]. As a conservative society, women in India at risk for or diagnosed with an STI, like HIV or HPV, are often perceived through a negative lens as these infections are societally linked to sexual misbehaviour and promiscuity [49, 50]. By engaging both men and women and creating safe spaces for open discussions and dialogue, topics related to sexual health can be normalized and hence de-stigmatized which are important steps in reducing cervical cancer mortality and morbidity [23].

The reduction in STI stigma in males, as the traditional head of the household in India, is an important step toward changing the biased attitudes toward women as men can be facilitators for women’s participation in screening. In our study, we found a significant reduction in stigma related to HPV among male participants following the SHEs. This was consistent with other educational initiatives which involved Nigerian men in women’s reproductive health discourses and reported an improvement in knowledge and reduction in HPV stigma in the community [19].

Higher uptake of HPV-SS confirmed our hypothesis that this method of screening is preferred over the traditional methods of screening. Almost all women and their supportive male partners, roughly 96%, accepted HPV-SS and chose this method of cervical cancer screening over other traditional methods. These results support findings from LMICs in Africa including Chad, Cameroon, and Kenya where the majority of women also had a higher acceptability of and preference for HPV-SS compared to other formats of screenings completed by healthcare providers [51–53]. In rural and Indigenous communities of South America, almost all participants were willing to complete the self-collection method and most of them also found it to be comfortable [54].

Although it is not yet widely implemented, HPV-SS provides considerable advantages to women across the globe, especially those living in rural or underserved areas who face limited access to cervical cancer screening and sexual health education. By completing the self-sampling test at home with informed educational tools and a support network, women can conveniently overcome limitations in accessing cervical cancer screening. Such limitations include lack of infrastructure for and travel time to health care services [55], cultural values and beliefs surrounding male healthcare providers and maintaining privacy of genitalia [56], as well the cost associated with fees for screening by a healthcare provider [57]. While such benefits exist, commonly cited barriers for self-sampling among women include a lack of self-confidence for collecting a reliable sample, fear of injuring oneself, concerns about accuracy of the test, and interpersonal stigma and burden related to diagnosis of sexually transmitted infections (STIs) [58–60]. As demonstrated by our findings, the widespread implementation of educational interventions that are tailored to various populations may address some of these factors.

There are a few limitations which should be taken into consideration when interpreting our results. First, the use of non-probability sampling methods (i.e., purposive sampling) may have introduced selection bias and diminished generalizability to the total population of rural Indian women. Although not ideal, the purposive sampling method was necessary because no sampling frame was available for this hard-to-reach women who were under or never screened for cervical cancer. Nevertheless, this study will provide the basis for future large-scale, nationwide studies that aim to assess knowledge, attitudes, stigma about cervical cancer and screening and acceptability of HPV-SS in rural communities. Second, the topic of cancer and sexual health are considered stigmatic among South Asian populations and our use of interview-based questionnaires may have influenced full disclosure of subject matter. This may have resulted in respondents’ tendency to give socially desirable responses. However, the use of ASHA workers who were considered trusted members of community, during recruitment phase of our study, helped to establish a good rapport between our medically trained staff who conducted the interviews and the study participants.

## Conclusions

Cervical cancer control rests on early detection through screening. Increasing knowledge and awareness of cervical cancer continues to remain a key challenge in LMICs. The lack of/limited knowledge about cervical cancer and screening that are reported in LMICs is an indication of violation of basic human rights in access to health and health information under Article 25 of the Universal Declaration of Human rights. Indeed, social inequalities are at the heart of many screening disparities.

Our project focused on UNS women in low-income rural areas of India where, access to health care services were limited, cervical cancer risk conditions were prevalent, and the general literacy rate and health literacy rate were low. Much of the gender-based differences were extensions of social and structural inequalities in terms of inequitable distribution of knowledge and resources. Taking into account the cultural, economic and political environment we found that cervical cancer screening uptake depends not only on women but also their male partners and male family members. Hence our approach in engaging men in the community directly impacted both women and men’s sexual health literacy by offering culturally and linguistically appropriate and gender-specific sexual health education. Considering the low literacy of our targeted communities, we utilized storytelling as a medium for sexual health communication. Our project aimed to reduce gendered stigma of cervical cancer as a sexually transmitted infections (STIs) through arts-based sextual health education. Stigma associated with STIs is an important barrier to women’s uptake of screening in LIMCs. Women are at risk of being stigmatized due to entrenched gender norms and stereotypes surrounding STIs. The SHEs offered to both men and women in our project an opportunity for open discussions on the misconceptions surrounding cervical cancer. By engaging men in sexual health education and stigma reduction we were able to facilitate and reduce gender biases and develop new gender norms that promoted uptake of cervical cancer screening among the most disadvantaged rural Indian women.

Moreover, our innovative and alternative method of screening (i.e., HPV-SS) empowered women by allowing them to undertake cervical cancer screening at a place and time that was convenient for them. Offering screening services and evidence-based technologies aligned with human rights that fit with the needs of hard-to-reach women have the highest potential to reduce the cervical cancer screening disparities. Having multiple screening methods and follow up strategies as was included in our study helped to promote screening coverage and continuity of care. The evidence gained from the study could be used to advance public health policies and inform scaling up of similar initiatives in other rural villages and states across rural India and other LIMCs.

## Data Availability

The datasets generated and/or analysed during the current study are available from the corresponding author on reasonable request.

## Abbreviations

ASHA: Accredited social health activists
HIV: Human immunodeficiency virus
HPV: Human papillomavirus
HPV-SS: Human papillomavirus Self-Sampling
LMICs: low- and middle- income countries
Pap test: Papanicolaou test
RA: Research Assistant
SHE: Sexual health education
STIs: Sexually transmissible/transmitted infections
UNS: Under or never screened
VIA: Visual inspection with acetic acid
